# Immunological signature of patients with thymic epithelial tumors and Good syndrome

**DOI:** 10.3389/fimmu.2022.908453

**Published:** 2022-08-18

**Authors:** Anna Maria Malfitano, Vittoria D’Esposito, Pietro De Placido, Marianna Tortora, Margaret Ottaviano, Erica Pietroluongo, Rocco Morra, Brigitta Mucci, Fabiana Napolitano, Liliana Montella, Mario Giuliano, Sabino De Placido, Daniela Terracciano, Giovannella Palmieri, Pietro Formisano

**Affiliations:** ^1^ Department of Translational Medical Sciences, University “Federico II”, Naples, Italy; ^2^ Research Unit (URT) Genomics of Diabetes, Institute of Experimental Endocrinology and Oncology, National Research Council, Naples, Italy; ^3^ Department of Clinical Medicine and Surgery, University Federico II, Naples, Italy; ^4^ Rare Tumors Coordinating Center of Campania Region (CRCTR), Naples, Italy; ^5^ Oncology Unit, Ospedale del Mare, Napoli, Italy; ^6^ ASL NA2 NORD, Oncology Operative Unit, “Santa Maria delle Grazie” Hospital, Pozzuoli, Italy

**Keywords:** thymic epithelial tumors, immunophenotype, T regulatory cells, Autoimmune diseases, chemokines, cytokines, growth factors

## Abstract

**Background:**

Thymic epithelial tumors (TETs) are frequently accompanied by Good Syndrome (GS), a rare immunodeficiency, characterized by hypogammaglobulinemia and peripheral B cell lymphopenia. TETs can be also associated to other immunological disorders, both immunodeficiency and autoimmunity.

**Methods:**

In this study, we enrolled TET patients with GS to address differences between patients with or without associated autoimmune diseases (AD). We analyzed the immunophenotype from peripheral blood of these patients focusing on selected immune cell subsets (CD4+T cells, CD8+T cells, T regulatory cells, NK cells, B-cells, monocytes, eosinophils, basophils, neutrophils) and serum levels of cytokines, chemokines and growth factors.

**Results:**

We observed higher number of leucocytes, in particular lymphocytes, B lymphopenia and lower number of T regulatory cells in TET patients with associated AD compared to TET patients without AD. In the group of TET patients with AD, we also observed increased serum levels of IL-15, VEGF, IP-10, GM-CSF, IL-6, and MIP-1α. Thus, we identified considerable differences in the lymphocyte profiles of TET patients with and without ADs, in particular a reduction in the numbers of B lymphocytes and T-regulatory cells in the former, as well as differences in the serum levels of various immune modulators.

**Conclusions:**

Although the pathogenic mechanisms are still unclear, our results add new knowledge to better understand the disease, suggesting the need of surveilling the immunophenotype of TET patients to ameliorate their clinical management.

## Introduction

Thymic epithelial tumors (TETs) are rare cancers originating from the thymus. The annual incidence of TETs is reported as 1.3 to 3.2 per million (1), with a mean age of diagnosis of 50-60 years. TETs are classified in thymoma, grouped into five types (A, AB, B1, B2 and B3), and thymic carcinoma (2). Current multimodality therapies (chemotherapy, surgery and radiotherapy) are reasonably effective in less advanced tumors (3). However, the rarity of these tumors highly delays the development of alternative therapeutic strategies and makes the management of TETs extremely challenging. TETs represent a particular type of malignancies as concerns clinical features, characterized by the singular combination with paraneoplastic syndromes, like autoimmune diseases (AD) (4) or immunodeficiency (5, 6). Among TETs, thymoma is the most prevalent, and is frequently associated with AD, followed by thymic carcinoma, which is instead rarely associated with AD. Most common AD associated with TETs are myasthenia gravis (MG), Graves’ disease, Hashimoto’s thyroiditis, aplastic anemia, pure red cell aplasia (PRCA), autoimmune hemolytic anemia, lichen planus pemphigus, systemic lupus and others. MG is frequently observed in thymoma patients showing an incidence up to 46% (7, 8). A multicenter retrospective study in TET patients reported PRCA as the most common type of cytopenia (30% of the cases) (9). Inflammatory bowel disease (IBD) and lichen planus have been reported as other autoimmune conditions (8, 10, 11). An open-label phase II trial aimed to address efficacy and safety of pembrolizumab in patients with TETs, reported as adverse events among others, MG (6.1%) and thyroiditis (3.0%) (12). The coexistence of thymoma with poli-autoimmunity such as rheumatoid arthritis (RA) and Hashimoto’s thyroiditis was described in a case report in which, the treatment with immunosuppressant improved swelling of joints and stiffness and reduced the tumor, thus suggesting autoimmune response as a common mechanism of these diseases (13). Indeed, a retrospective search of surgical database for patients operated for thymoma revealed inter-relationship between thymomas and MG or other autoimmune syndromes (14). In a retrospective study on 260 patients with thymoma, Isaac’s syndrome was reported as the second most frequent AD after MG (15). This incidence was confirmed in another study (3.5%) reporting no risk factor for developing AD after thymectomy (16). Thymectomy is recommended for all MG patients with thymoma, however, its impact on the outcome of other AD is variable, and in some cases, might worsen the evolution of the disease (17, 18).

A less frequent and under-evaluated parathymic syndrome, in the spectrum of autoimmunity and immunodeficiency is Goodʼs syndrome (GS) (19, 20). GS displays hypogammaglobulinemia, low or absent B cells, abnormal CD4/CD8 T cell ratio and compromised T cell response to mitogens. Patients with GS frequently undergo fungal, bacterial and opportunistic infections due to humoral and cell-mediated immunodeficiency and their management is quite challenging because of the sequelae of tumor-related therapies and life-threatening problems (6). An observational study suggested that chemotherapy administered to TET patients could be adopted also in patients with GS and/or immune cytopenia but required a very accurate monitoring. The authors highlighted the relevance of screening for immunological characteristics of GS patients with autoimmune cytopenia before the administration of immunosuppressive treatments. In this context, the evaluation of immunoglobulin titers, B cell counts and CD4+ T lymphocyte phenotyping is mandatory (9). Notably, the mortality rate of these patients receiving immunoglobulin replacement therapy, was reduced compared to previous studies (10, 21, 22); the deaths reported in advanced tumors were ascribed to frequent infections likely occurring for the several courses of chemotherapy (9).

The increased prevalence of AD and immunodeficiency in TET patients is supportive of cell-mediated immune defect. Some abnormalities that may influence normal T-cell development have been detected in thymomas. In particular, a distorted tumor architecture and low expression of MHC class II on neoplastic cells was observed as well as the absence of the autoimmune regulator (AIRE) gene and a reduced production of T -regulatory (Treg) cells.

Studies trying to address the immunological mechanisms responsible of these findings in thymoma, suggested aberration of T cell subsets and cytokines (23). In patients with thymoma and GS, a progressive decline of B, CD4+T and natural killer (NK) cells was observed (24). Defect of Treg cells in thymoma patients has been investigated in thymoma-associated MG, suggesting a Treg cell role in MG development (25–28). In this study, we addressed differences in immune cell phenotype and serum levels of a panel of cytokines, chemokines and growth factors in TET patients with and without AD.

## Materials and methods

### Patients

Patients recruited at the Rare Tumors Coordinating Center of Campania Region (CRCTR), of Federico II of Naples from June 2019 provided informed consent for their blood sample collection. Thymus neoplasia was rated in agreement with the World Health Organization (WHO) classification and tumor stage was determined according to the Masaoka staging system (29). Patients donating blood had been for at least 3 weeks treatment-free. For the aims of this study, patients with TETs were divided in two groups: patients with only GS (12 patients) and patients with GS plus AD (TET-AD, 17 patients).

### Blood cell count

The blood collected from patients was processed for routine hematology. Laboratory data were used to obtain white blood cell (WBC) counts, absolute lymphocyte, neutrophil, monocyte, eosinophil and basophil counts. These parameters were compared to the normal range in **Table 1**.

**Table 1:** Parameters of blood cell counts in TET and TET-AD patients vs. normal range values.

Parameter	TET TET-AD	Normal range
WBC (x10^3^/µl)	6.0 (3.1-9.3) 9.0 (2.9-15.0)	4.0-10.8
Neutrophils (x10^3^/µl)	4.1 (1.8-7.8) 5.9 (0.6-11.8)	1.9-8.0
Lymphocytes (x10^3^/µl)	1.2 (0.9-2.1) 2.1 (0.9-4.8)	0.9-5.2
Monocytes (x10^3^/µl)	0.4 (0.2-0.8) 0.5 (0.3-0.9)	0.16-1.0
Eosinophils (x10^3^/µl)	0.1 (0.0-0.3) 0.07 (0.0-0.3)	0.0-0.8
Basophils (x10^3^/µl)	0.04 (0.0-0.1) 0.04 (0.0-0.1)	0.0-0.2

The table displays for each hematological parameter reported the means and the minimum and maximum value for TET and TET-AD patients. The normal control range values are also reported.

### Immunofluorescent staining and lysis of whole blood

Peripheral blood from patients was processed for immunophenotyping, according to the 8-color immunophenotyping kit and Treg detection kit (CD4/CD25/CD127) instructions (MACS, Miltenyi biotec). Whole blood (100µl) from each patient was stained with 10 µl of 8-color immunophenotyping cocktail containing fluorochrome-conjugated antibodies: anti-CD3 conjugated to PE, anti-CD4 conjugated to VioBright 667, anti-CD8 conjugated to APC-Vio 770, anti-CD14 conjugated to VioBlue, anti-CD16 conjugated to VioBright 515, anti-CD19 conjugated to PE-Vio 770, anti-CD45 conjugated to VioGreen, anti-CD56 conjugated to VioBright 515. Additionally, 10µl of 7-AAD staining solution were added to each tube. Stained blood was incubated 10’ in the dark at room temperature (RT). After the incubation, red blood cell lysis solution (2 ml, 1X) was added to each tube and immediately vortexed thoroughly for 3 seconds and incubated for 15’ in the dark at RT. Solution was aspirated after centrifugation at 300g for 10’. Cell pellet was re-suspended in a suitable amount of buffer and immediately subjected to by flow cytometry (BD LSRFortessa, BD Biosciences, San Jose, CA, USA), analyses were performed using Flowlogic Software (MACS, Milteny Biotech). Similar procedure was adopted after staining the whole blood (100 µl x tube) with Treg detection staining cocktail containing fluorochrome-conjugated antibodies: anti-CD4 conjugated to FITC, anti-CD25 conjugated to APC, anti-CD45 conjugated to Vio Blue, anti-CD127 conjugated to PE.

### Quantification of cytokines, chemokines, and growth factors

Serum samples were screened to quantify human cytokines, chemokines, and growth factors with pre-formed kits by Bioplex multiplex (Bio-Rad, Hercules, CA, USA, cat # M500KCAF0Y). Samples were diluted (1:4) and 50µl were used, according to the manufacturer’s instructions. The concentration of IL-1RA, IL-1β, IL-2, IL-4, IL-5, IL-6, IL-7, IL-8, IL-9, IL-10, IL-12(p70), IL-13, IL-15, IL-17A, basic fibroblast growth factor (FGF), eotaxin, granulocyte-colony stimulating factor (G-CSF), granulocyte-macrophage colony-stimulating factor (GM-CSF), interferon-γ (IFN-γ), interferon-γ inducible protein 10 (IP-10), monocyte chemoattractant protein-1 (MCP-1), macrophage inflammatory protein-1 (MIP-1) α, MIP-1β, C–C motif chemokine ligand 5 (CCL5)/RANTES, TNF-α, platelet-derived growth factor (PDGF-BB) and vascular endothelial growth factor (VEGF) were determined according to the manufacturer’s protocol as previously described (30). The magnetic bead-based assay was performed on a Bio-Plex 200 System (Bio-Rad, Hercules, CA, USA).

### Statistical analyses

Results are presented as mean values ± standard deviation (SD). Statistical analyses were performed using GraphPad 8.0 software (GraphPad Software Inc., La Jolla, Ca). D’Agostino-Pearson normality test was used to evaluate whether the continuous data were normally distributed, and a two-tailed t-test for independent samples was used. Alternatively, we used Mann-Whitney test for non-parametric analysis. Grouped data were analyzed by Two-way Anova. Detected outliers were removed according to ROUT method with Q coefficient 1%. p values <0.05 were considered statistically significant.

## Results

### Background characteristic of patients

Clinical features of TET-GS patients with and without AD are detailed in **Table 2**, as regard of age, sex, thymectomy, tumor histology and stage, MG and other AD, second primary cancer, incidence of infections.

**Table 2:** Clinical features of TET-GS patients with or without AD (n 29). .

Characteristic	TET (*N* = 12)	TET-AD (*N* = 17)
Age (years)		
** Mean (median)**	53.75	60.82
** Range**	45–65	38–75
**Sex, *n* (%)**		
** Male**	8 (67)	5 (29)
** Female**	4 (33)	12 (71)
**Thymectomy, *n* (%)**		
** Yes**	4 (33)	16 (94)
** No**	8 (67)	1 (6)
**Histological type, *n* (%)**		
** Thymoma**	10 (83)	17 (100)
** A**	0	6 (35%)
** A B**	0	4 (24%)
** B1**	0	1 (6)
** B2**	2 (17)	2 (12)
** B1–B2**	0	0
** B2–B3**	1 (8)	1 (6)
** B3**	7 (58)	3 (18)
** Not otherwise specified**	0	0
** Thymic carcinoma**	2 (17)	0
**Stage of disease according to TNM staging, *n* (%)**		
** I**	0	3 (18)
** II**	0	2 (12)
** IIIA**	2 (17)	0
** IIIB**	0	0
** IVA**	5 (42)	4 (24)
** IVB**	5 (42)	8 (47)
**Myasthenia gravis ,, *n* (%)**		
** Yes**	0	9 (53)
** No**	12 (100)	8 (47)
**Other autoimmune disorders, *n* (%)**		
** PRCA**	0	5 (29)
** Cholitis**	0	6 (35)
** Arthritis**	0	3 (18)
** Thyroiditis**	0	4 (24)
** Other**	0	2 (12)
** Polyautoimmunity**	0	7 (41)
**Second primary cancer, *n* (%)**		
** Kaposi’s sarcoma**	0	6 (35)
** Prostate**	0	1 (6)
** Bronchial**	1 (8)	0
** Other**	0	1 (6)
**All type of infection, *n* (%)**	12 (100)	16 (94)
**Typical site: sinopulmonary, *n* (%)**	10 (83)	14 (82)
**Pathogen, *n* (%)**		
** Bacteria: *Streptococcus pneumoniae***	5 (42)	6 (35)
** Virus: cytomegalovirus**	4 (33)	11 (65)
** Fungus: *Candida***	6 (50)	9 (53)

The table reports the groups of TET patients with GS (12 without AD and 17 with AD) along with their clinical characteristics. TMN, tumour, node, metastasis.

### Quantification of leucocytes by blood cell count

**Figure 1:**
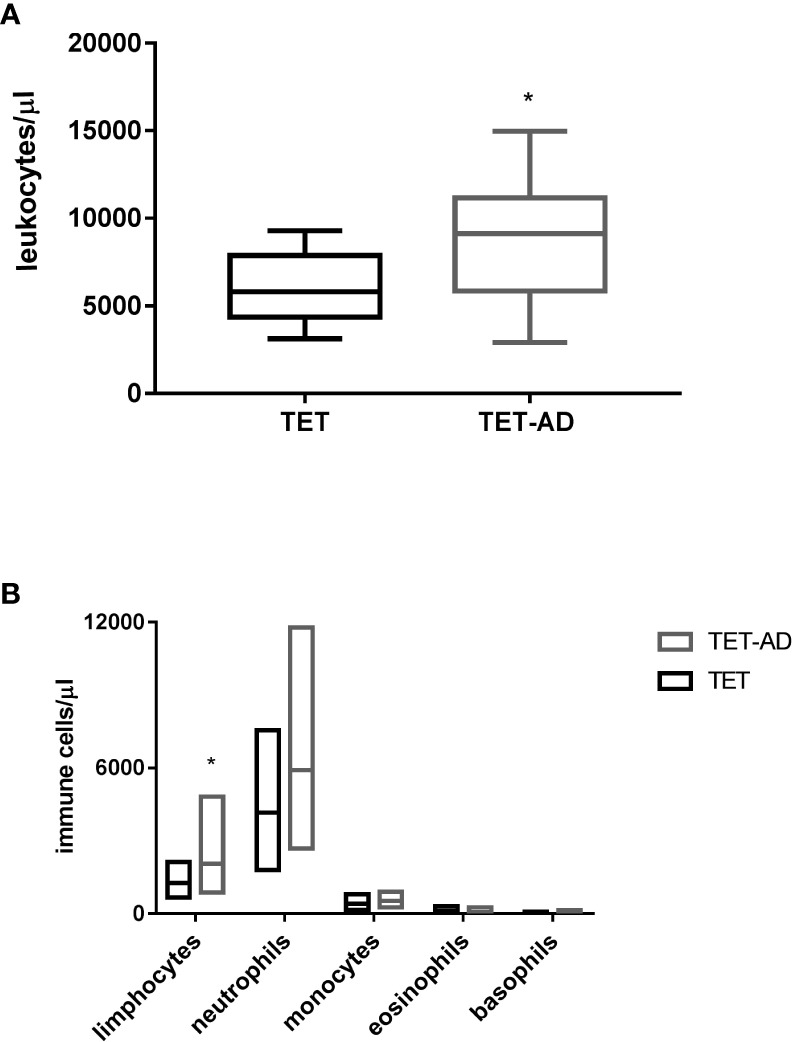
Leucocyte and immune cell subset counts in thymoma patients. Blood samples collected from patients with TETs and TET-AD, both with GS, were processed for blood cell count analysis. The number of leucocytes **(A)** and the number of lymphocytes, neutrophils, monocytes, eosinophils and basophils **(B)** expressed in leucocytes/µl and immune cell/µl respectively is reported for both groups of patients. Results are represented as mean values ± SD in box plots. Unpaired parametric two tailed t test was performed for the analyses of leucocytes, neutrophils, monocytes and basophils. Mann-Whitney test was used for the analyses of lymphocytes and eosinophils *p < 0.05.

The number of WBCs was determined from blood cell count. We observed in TET-AD patients a higher number of leucocytes (9021,3 ± 3658,32 in 15 patients) with respect to TET patients (6010 ± 1966,51) as reported in **Table 1**, and the increase was statistically significant (**Figure 1A**). In order to assign the increase observed in total leucocytes to a particular cell population, we reported also the number of lymphocytes, neutrophils, monocytes, eosinophils and basophils. The contribution of lymphocytes to the increased number of leucocytes in TET-AD group was statistically significant, whereas in other cell populations no difference was observed between the two groups of patients (**Figure 1B**; **Table 1**).

### Identification and quantification of immune cell populations by flow cytometry

**Figure 2:**
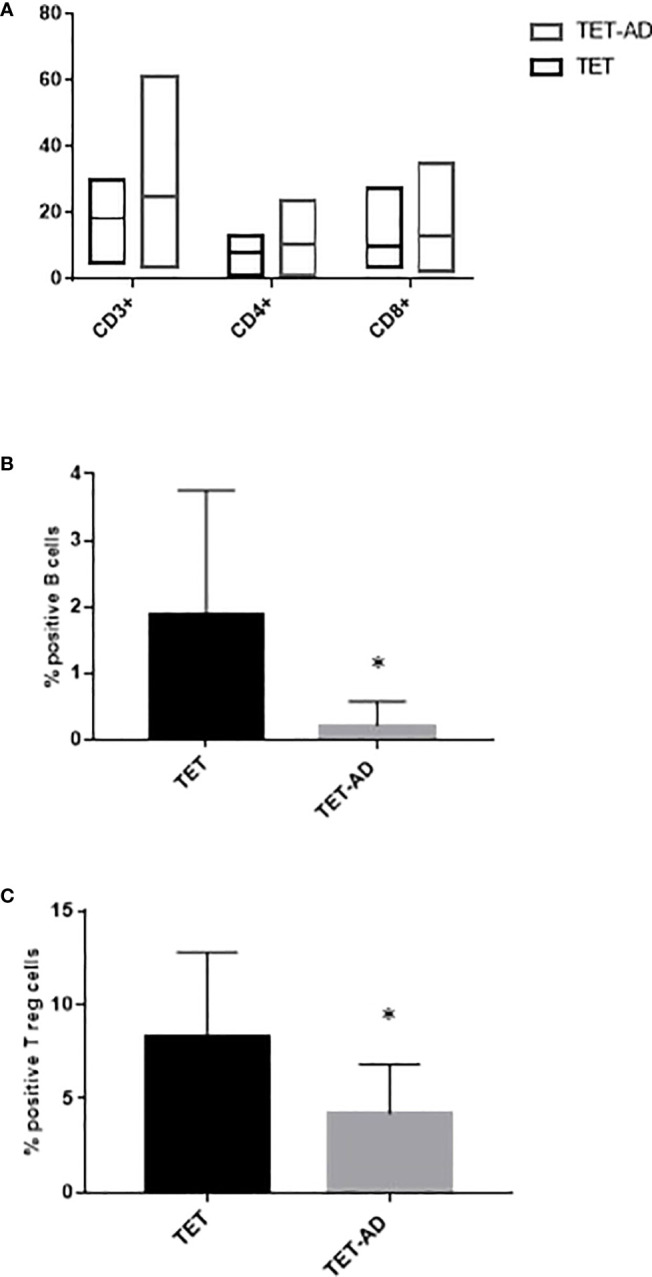
Flow cytometry profile of immune cell subsets. Immunophenotype of T cells, CD3+, CD4+, CD8+ **(A)**, B cells **(B)** and Treg cells **(C)** is reported for TET and TET-AD patients. Results are represented as mean values ± SD. The statistical analysis was performed by Mann-Whitney test *p < 0.05.

The increase of the number of lymphocytes observed in TET-AD patients (**Figure 1**), prompted us to investigate potential difference between the two groups of patients in the percentage of lymphocyte subsets: T, (CD3+, CD4+, CD8+), B (CD19+) and natural killer (NK, CD56+). First, we selected live leukocytes, based on CD45 immunodetection, separated from debris via forward scatter (FSC) and side scatter (SSC). On live leukocytes, immune cell subsets were identified by gating strategies. The sentence can be: We observed a slight increase of T cell subsets (CD3+, CD4+, CD8+ T cells) in TET-AD patients compared to TET patients although differences did not reach statistical significance, similar values of CD4/CD8 ratio, normally greater than 1 (31) in TET and TET-AD patients were also detected: 0.91 ± 0.13 and 0.95 ± 0.09 (mean ± SEM), respectively. The reduced CD4/CD8 ratio compared to the normal range is consistent with the slight increase of CD8+ versus CD4+ T cells observed in both the groups, although the differences were not statistically significant. We observed a decrease of B cells, whose levels are much lower compared to T lymphocytes, in TET-AD patients with respect to TET patients without AD (**Figure 2B**). The analyses of NK cells revealed no difference between the two groups of patients with means ± SEM of 4.68 ± 1.07 and 4.50 ± 0.78 in TET and TET-AD patients respectively (data not shown). Monocytes were distinguished, based on CD14 surface marker and further divided into classical, intermediate and non-classical monocytes by CD16 expression. Neutrophils and eosinophils were distinguished based on CD16^+^/SSC^high^ and CD16^-^/SSC^high^ populations. However, the analyses of monocytes, neutrophils and eosinophils did not show any significant difference between TET and TET-AD patients (not shown). Treg cells, evaluated on CD4+T cells, were identified gating CD25^high^CD127^dim/neg^ surface markers. Treg cells (**Figure 2C**), were down-regulated in TET-AD patients. A normal range of Treg cells previously evaluated on healthy individuals by CD25^high^CD127^dim/neg^ markers reported a variation from 6-8% of circulating CD4+ T cells (32). In our data the values obtained (**Figure 2C**) report means ± SEM of 8.31 ± 1.29 and 4.21 ± 0.64 in TET and TET-AD patients respectively. Thus, the value of Treg cells we observed in TET-AD patients is under the limit of the normal range.

### Analysis of circulating levels of cytokines, chemokines, and growth factors

**Figure 3:**
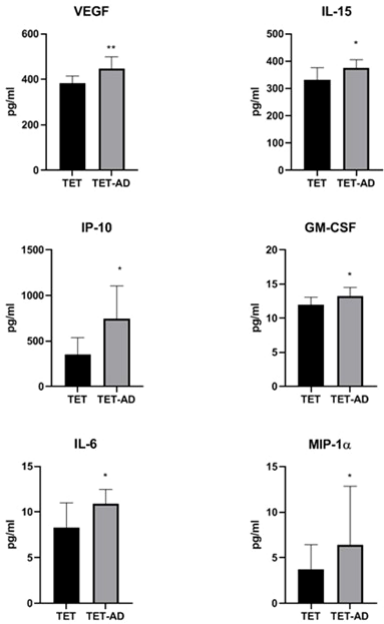
Serum concentration of cytokines, chemokines and growth factors. Histograms indicate cytokine concentrations (pg/ml) in TET (N = 8) and TET-AD (N = 9) patients. The histogram of IL-6 includes TET (N = 7) and TET-AD (N = 9) patients. The histogram of MIP-1α includes TET (N = 7) and TET-AD (N = 8) patients. Results are indicated as mean ± SD. A two-tailed t-test for independent samples test was performed to analyze data (*p < 0.05; **p < 0.01).

The analysis of a panel of cytokines, chemokines, and growth factors (**Table 3**) revealed a statistically significant increase of circulating levels of VEGF, IL-15, IP-10, GM-CSF, IL-6 and MIP-1α in TET-AD patients compared to TET patients (**Figure 3**). Comparable levels of IL-1RA, IL-1β, IL-2, IL-4, IL-5, IL-6, IL-7, IL-8, IL-9, IL-10, IL-12(p70), IL-13, IL-17A, b-FGF, eotaxin, G-CSF, IFN-γ, MCP-1, MIP-1β, CCL5/RANTES, TNF-α and PDGF-BB between the two groups of patients were observed.

**Table 3:** Serum concentration of cytokines, chemokines, and growth factors.

Secreted factors	TET	TET-AD	p-value
**IL-1β**	1.9 ± 0.5	2.3 ± 0.4	0.131
**IL-1RA**	304.6 ± 151.4	309.9 ± 52.1	0.922
**IL-2**	14.9 ± 3.1	16.9 ± 1.6	0.109
**IL-4**	6 ± 2.8	7.5 ± 2.3	0.26
**IL-5**	51.8 ± 15.4	65.6 ± 11.8	0.056
**IL-6**	8.2 ± 2.7	10.8 ± 1.6	*0.031*
**IL-7**	43.2 ± 8.4	47.5 ± 7.1	0.281
**IL-8**	22.7 ± 8.8	23.4 ± 4.2	0.853
**IL-9**	242 ± 81.5	249.72 ± 24.4	0.792
**IL-10**	23.9 ± 9.5	30.8 ± 5	0.077
**IL-12**	11.2 ± 1.5	12.5 ± 2.5	0.245
**IL-13**	4.8 ± 1.6	6.1 ± 1.8	0.14
**IL-15**	331.8 ± 44.4	374.9 ± 30.6	*0.032*
**IL-17**	26.7 ± 9.4	33.8 ± 7.6	0.106
**EOTAXIN**	64.5 ± 54.4	93.1 ± 52.9	0.289
**b-FGF**	75.2 ± 16.8	82.9 ± 9.4	0.257
**G-CSF**	351.2 ± 192.7	427.4 ± 70.3	0.311
**GM-CSF**	11.9 ± 1	13.2 ± 1.2	*0.042*
**IFN-γ**	12.2 ± 2.9	12.8 ± 1.5	0.602
**IP-10**	354.3 ± 183.8	748.7 ± 357.5	*0.013*
**MCP-1**	30.9 ± 19.8	34.4 ± 14.3	0.68
**MIP-1α**	2.8 ± 0.5	4.2 ± 1.3	*0.017*
**MIP-1β**	77.4 ± 33.8	88.8 ± 19.6	0.403
**PDGF**	1029.3 ± 483	1082.1 ± 435.9	0.821
**RANTES/CCL5**	6987.4 ± 4178.3	8580.8 ± 2144.4	0.329
**TNF-α**	44.6 ± 17.3	52.2 ± 3.6	0.246
**VEGF**	383.2 ± 31.9	448.2 ± 50.9	*0.007*

Concentrations are expressed as pg/ml. Results are indicated as mean ± SD. We indicated in italics p values <0.05.

## Discussion

Immunodeficiency and autoimmunity were previously described as combined features in patients with thymoma (33, 34) highlighting a poor outcome (with the exclusion of MG) of these patients (23). In a previous study, we characterized immunophenotype and T cell repertoire in a cohort of 30 thymoma patients. We observed that GS development was accompanied by a progressive reduction of B, CD4+ and NK lymphocytes, modifications paired with naïve CD8+ CD45+RA+T cell subsets (24). Accumulation of naïve CD8+ CD45+RA+T cells is assumed as a hallmark of thymoma and has been reported to be more severe in subgroup of patients with MG (35). Consequently, GS patients often display an inverse CD4+/CD8+ ratio, CD4+ T cell lymphopenia, and impaired T cell mitogenic response (22). In the present study, we investigated the immunological abnormalities in patients with TETs associated with GS in the presence or in the absence of autoimmunity, which GS is the most common manifestation observed in these patients. The increased number of leucocytes in the TET-AD group of patients supports a profile of autoimmunity. In particular, we detected that lymphocytes represent the immune cell population accounting for leucocyte increase in TET-AD patients because no increase in other cell subsets (neutrophils, monocytes, eosinophils, basophils) was observed. The total number of lymphocytes accounts for T, B and NK cells. However, since B cells are scarcely present in TET-AD patients and NK cells are low in percentage and not different between TET and TET-AD patients, we speculated that the increase in the absolute number of lymphocytes might be due to the T cell compartment. However, the percentage of CD3+, CD4+ and CD8+ T cells although slightly increased in TET-AD patients compared to TET patients, did not reach the statistical significance. B lymphopenia typically observed in patients with clinical signs of GS immunodeficiency, was more marked in TET-AD than in TET patients.

We previously suggested that the oligoclonal expansion of CD8+T cells reported in the bone marrow of GS patients might mediate B cell precursor killing and consequently peripheral B cell loss (24). In our cohort of TET-AD patients, these mechanisms might be exacerbated by enhanced peripheral CD8+T cell activity, thus explaining the discrepancy between the two groups of patients in B lymphopenia. This hypothesis is supported by a study in a mouse model demonstrating that CD8+T cells primed by cytokines of innate immune response such as IL-15, of which we detected an increase in TET-AD patients, display enhanced responsiveness to antigens/autoantigens or to weakly agonistic TCR ligands. The results of this study suggested a role of inflammatory cytokines in triggering autoreactive CD8+T cells (36). Indeed, Treg cell percentage was lower in TET-AD patients compared to TET patients. Treg cells play a fundamental role in the maintenance of self-tolerance and controlling autoimmunity. It was suggested that Treg cell decrease in the thymic tissues of patients with thymoma might trigger the development of MG and our data seem to support this hypothesis. Indeed, neoplastic transformation in thymoma possibly has a role in the abnormal processes of positive and negative selection of thymocytes that guide to the loss of self-tolerance and increase of autoreactive T lymphocytes in the peripheral blood (7). Changes in Treg cell number and distribution in the peripheral blood of thymoma patients with MG was extensively investigated. However, no conclusive data were reached.

In this context, our analyses of a large panel of cytokines, chemokines, and growth factors highlighted the modulation of IL-15, IL-6, VEGF, IP-10, GM-CSF and MIP-1α with differences observed between the two groups of patients. In particular, we detected higher levels of all these factors in TET-AD than in TET patients.

Il-15 is a pleiotropic pro-inflammatory cytokine endowed with numerous biological functions (37), ranging from T cell (38), neutrophil and macrophage activation to a critical role in dendritic cell activity in several systems (37). In various AD, increased levels of IL-15 in inflamed tissues and in the circulation were reported, likely contributing to AD pathogenesis (39). Additionally, previous studies described the key role of this cytokine in MG pathogenesis, suggesting that its release by muscle cells (39), worsened inflammation and the clinical course. Our data are in agreement with above reported studies and suggest that the increase of IL-15 in TET-AD patients might sustain T cell enhanced population and autoimmunity. Indeed, a study suggested that IL-15 enhanced effector T cell activity in the presence of Treg, and although functional in Treg, did not modify their inhibitory function (40). In multiple sclerosis, an AD with inflammatory component, IL-15 was reported to potentiate CD8+T cell activity (41) and promote their production of GM-CSF (42).

IL-6 plays various role in AD, its abnormal regulation or over-secretion can lead to the occurrence of AD. IL-6 was demonstrated to contribute to the development of autoreactive CD4+T cell responses by suppressing the induction of Treg cells (43).

IL-6 regulates the balance between Th17 and Treg cells, for example, in experimental autoimmune MG (EAMG), the treatment of myasthenic rats with neutralizing anti-IL-6 antibodies shifted the equilibrium in favor of Treg cells with consequent arrest of EAMG (44). A very recent study demonstrated increased levels of IL-6 in patients with MG and thymoma compared to patients with thymoma alone, thus suggesting its association with MG and its potential role in Treg cell decrease (45). Indeed, IL-6 was also reported to inhibit Treg cell differentiation induced by TGF-β (46) Our results showing increased IL-6 serum levels in TET-AD patients compared to TET patients, support the role of this cytokine in sustaining AD and contributing to Treg cell decrease. A cytokine profile of GS patients is not available, however, it was suggested that recurrent respiratory infections suffered by these patients could be related to the intracellular expressions of IL-17A and IFN-γ (47).

The role of VEGF in supporting the growth of some thymomas was suggested in a study reporting a correlation between VEGF receptor (VEGF-R) in endothelial and epithelial cells and the levels of VEGF (48). In a previous report, no differences in VEGF levels were detected between patients with MG and thymoma and patients with MG without thymoma, however, the increased levels of VEGF observed suggested its potential role in the pathogenesis MG (39). Our data support this hypothesis highlighting the increase of VEGF levels in TET-AD patients with respect to TET patients without AD.

The increase of IP-10 in TET-AD patients is consistent with the role of this chemokine in AD pathogenesis. IP-10 detected at high levels in peripheral fluids is a marker of host immune response, particularly T helper (Th)1 orientated. In particular, the enhanced production of IFN-γ and TNF-α stimulates IP-10 secretion by various cells perpetuating the autoimmune process (49).

GM-CSF is involved in the inflammatory context of many AD. GM-CSF was observed in the peripheral blood of RA patients and its production by CD4+ cells was related to Th1 activation and IL-15 (50). Indeed, GM-CSF was reported to mediate autoimmunity by enhancing IL-6–dependent survival of antigen specific CD4+ T cells and promoting generation and maintenance of Th17 cells *in vivo* (51). We observed increase of GM-CSF in TET-AD patients with respect to TET patients, consistently with its role in AD.

MIP-1α is a pro-inflammatory chemokine secreted by various types of immune cells upon activation and plays important roles in cell recruitment, trafficking and inflammatory responses. MIP-1α also emerged as a prognostic biomarker in both solid and hematological malignancies (52). The increased levels of MIP-1α detected in TET-AD patients are in agreement with the increased amount of leukocytes and lymphocytes observed in this group of patients.

The exclusion of AD contribution or a control for AD might be a limitation in our study. However, we have to consider that a proper AD control should require a not compromised B cell evolution like that reported in our patients, since it is accepted a collaboration of T cells and B cells in driving autoimmunity. Indeed, the recurrent infections in these patients make unique their immune system and difficult to compare with a “normal” immune system with AD. It was reported a multifaceted relationship between infections and autoimmunity. In particular, it was suggested that pathogens can shift the balance Th1-Th2 towards an immunosuppressive status, or infections in other locations might recall autoreactive cells preventing them from reaching and destroying the sites of autoimmunity (53).

**Figure 4:**
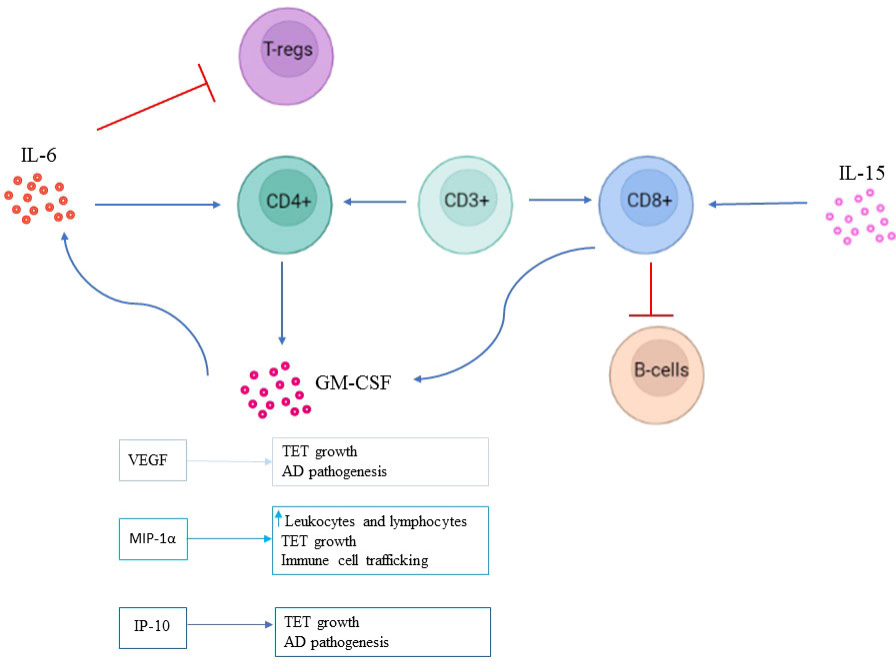
Potential mechanism in TET-AD patients. The presence of CD3+T cells is influenced by pro-inflammatory cytokines able to regulate CD4+ and CD8+ T cell function in the context of TETs with GS and AD. IL-6 is known to promote autoreactive CD4+ T cells and inhibit Treg cells. We propose that IL-6 elicits both the reported functions in TET-AD patients enabling CD4+ T cells to secrete GM-CSF known to enhance IL-6–dependent survival of CD4+ T cells. IL-6 might contribute to the decrease of Treg cells observed in TET-AD patients. On the other hand, IL-15 might support CD8+T cell function favoring its inhibitory effects on B cell development and likely inducing the secretion of GM-CSF by CD8+T cells that further sustains IL-6 activity. VEGF and IP-10 might exert a role in TET growth and in AD pathogenesis, whereas MIP-1α might be also involved in TET growth, and in the observed increased numbers of leukocytes and lymphocytes and in immune cells trafficking.

In conclusion, our study reports a profound remodeling in the B and T lymphocytes, highlighting differences in the Treg cell subset between TET-AD and TET patients. Additionally, secreted factors, cytokines and chemokines sustain the AD process. Although the pathogenic mechanism is still undefined, our data contribute to a better knowledge of the disease and suggest the need of surveilling the immunophenotype of these patients to ameliorate the clinical management, as summarized in **Figure 4**. Moreover, our study suggests that the identified immuno-profiles of patients with both thymic tumors and AD resemble those of TET patients responding to immune-checkpoint inhibitors, with relative high toxicity. The clinical management of patients with TETs and GS is still challenging and matter of debate. In case of coexisting AD, considering the greater decrease of B lymphocytes, the physician should carefully consider the management of immunosuppressive treatments in patients with TETs, thus we believe that a multidisciplinary approach is mandatory.

## Data availability statement

The original contributions presented in the study are included in the article/supplementary material. Further inquiries can be directed to the corresponding authors.

## Ethics statement

The studies involving human participants were reviewed and approved by Comitato Etico Università degli studi di Napoli Federico II, Dipartimento di Medicina Pubblica e della Sicurezza Sociale - Sezione di Medicina Legale - Edificio 20 - in *Via* S. Pansini 5. The patients/participants provided their written informed consent to participate in this study.

## Author contributions

AM carried out the experiments, analyzed the data and drafted the manuscript. VD carried out the experiments and analyzed the data. PD, MT, MG, EP, RM, BM, MO, LM contributed to patient collection and clinical data analysis. FN carried out the experiments, MG, SD and DT interpreted the data and provided critical feedback. GP and PF planned and supervised the work. All authors contributed to the article and approved the submitted version.

## Funding

This research was funded in part by the Regione Campania POR FESR 2014–2020–Objective 1.2.—Realization of Technology Platform to fight oncologic diseases (RARE PLAT NET, SATIN, and COEPICA Projects) and by the Italian Association for the Cancer Research—AIRC (grant IG19001) to PF.

## Acknowledgments

This study was part of the research activity of the Rare Tumors Coordinating Center of Campania Region (CRCTR), recognized as full member of the European Reference Network (ERN-EURACAN).

## Conflict of interest

The authors declare that the research was conducted in the absence of any commercial or financial relationships that could be construed as a potential conflict of interest.

## Publisher’s note

All claims expressed in this article are solely those of the authors and do not necessarily represent those of their affiliated organizations, or those of the publisher, the editors and the reviewers. Any product that may be evaluated in this article, or claim that may be made by its manufacturer, is not guaranteed or endorsed by the publisher.

## Abbreviations

TETs, Thymic epithelial tumors; AD, autoimmune diseases; MG, myasthenia gravis; GS, Goodʼs syndrome; AIRE, autoimmune regulator; Treg, T-regulatory; IL, interleukin; FGF, fibroblast growth factor; G-CSF, granulocyte-colony stimulating factor; GM-CSF, granulocyte-macrophage colony-stimulating factor; IFN-γ, interferon-γ; IP-10, interferon-γ inducible protein 10; MCP-1, monocyte chemoattractant protein-1; MIP-1, macrophage inflammatory protein-1; CCL5, C–C motif chemokine ligand 5; PDGF, platelet-derived growth factor; VEGF, vascular endothelial growth factor; EAMG, experimental autoimmune MG; Th, T helper.
